# Do cognitive distortions explain the longitudinal relationship between life adversity and emotional and behavioural problems in secondary school children?

**DOI:** 10.1002/smi.2743

**Published:** 2017-02-15

**Authors:** Constantina Panourgia, Amanda Comoretto

**Affiliations:** ^1^ Centre for Behavioural Change, Department of Psychology, Faculty of Science & Technology Bournemouth University Poole UK; ^2^ Department of Health and Social Care London South Bank University London UK

**Keywords:** cognitive distortions, emotional and behavioural problems, life adversity, mediator, structural equation models

## Abstract

Research has shown that children exposed to life adversity are at higher risk of negative developmental outcomes than those enduring lower stress levels. Life adversity can lead, among other things, to emotional and behavioural problems. Several factors have been studied to explain this relationship, with several investigators underlining the role of thought structures such as cognitive distortions, which refer to negatively biased information‐processing of external events. This can help explain why some individuals characterised by adverse personal life stories interpret ambiguous events in a negatively biased way. This study was aimed at assessing the mediating role of cognitive distortions in the longitudinal relationship between life adversity and two dimensions of psychopathology, namely, emotional and behavioural problems in 247 secondary school children attending three state secondary schools in one county in the South East of England. An increase in life adversity was associated with an increase in cognitive distortions, which was in turn related to a higher number of symptoms reflecting behavioural issues. In terms of practical applications, an effort to protect children from further exposure to adverse life events could represent a step forward to prevent the development of future behavioural problems in at‐risk children.

## 1 INTRODUCTION

The relationship between life adversity and emotional and behavioural problems in childhood and adolescence has long been established by a number of studies (Grant, Compas, Thurm, McMahon, & Gipson, 2004 for a review). There are several ways, based on frequency, duration, nature or severity, to assess life adversity. Consistent with previous investigations (e.g., Luthar, 1991; Tiet et al., 1998, 2001) this study examined life adversity by using a self‐report checklist to measure the experience of “objective” environmental conditions over which young people had little or no control (e.g., someone in the family had been arrested). The impact of chronicity of life adversity on outcomes was also taken into account. Bronfenbrenner's (1989) ecological theory, describing risk as stemming from various social domains, was also used as a framework to investigate life adversity in the child (e.g., school change), family (e.g., a negative change in parents' financial situation), and peer context (e.g., a seriously ill or injured close friend). Finally, in line with the cumulative risk approach to risk modelling (Rutter, 1979), this study considered the exposure impact of cumulative life adversity (in other words, the number of events experienced by a child over a specified period) to investigate the effect of life adversity accumulation on child outcomes.

The impact of life adversity, especially when prolonged and severe, can be pervasive and may affect many areas of a child's life (Evans & Pilyoung, 2013). There is a significant literature suggesting how children experiencing life adversity are at higher risk of maladjustment than their peers not experiencing the same number of difficulties. This has the potential to lead to a variety of negative consequences, such as academic underachievement, low self‐esteem, school maladaptive functioning, physical problems, and troubles in social relationships (Werner, 2013). Empirical evidence also exists for the relationship between life adversity and anxiety (Allen, Rapee, & Sandberg, 2008), depression (Mitchell, Tynes, Umaña‐Taylor, & Williams, 2015), substance abuse (Dube et al., 2003), eating (Johnson, Rohan, & Kirk, 2002), and conduct disorders (Green, Russo, Navratil, & Loeber, 1999), as well as aggressive behaviours (Mitchell et al., 2015).

Numerous factors have been studied to explain the relationship between life adversity and psychopathology, and many investigations of stressful life events have emphasised the key role of cognitive structures. This study focused on cognitive distortions, also called negative cognitive errors, which correspond to a typology of cognitive structure whose roots lie in Beck's (1976) cognitive model of depression. This theory is generally used to describe the process by which the individual interprets external events in a negatively biased way.

According to Beck, Rush, Shaw, and Emery (1979), there are seven types of cognitive distortions: (a) *selective abstraction* (attending to negative aspects of experiences in a selective way), (b) *overgeneralisation* (believing that a negative outcome will happen in similar situations in the future), (c) *catastrophisation* (always thinking of the worst on the premise that the worst is most likely to happen), (d) *personalisation* (inappropriately attributing the cause of external events to oneself), (e) *temporal causality or predicting without sufficient evidence* (believing that something negative that happened in the past is likely to also occur in the future), (f) *self‐reference* (believing that oneself, especially one's bad performance, is the centre of everyone's attention), and (g) *dichotomous thinking* (only focusing on the extreme results of a situation, be that a positive or negative one).

According to Beck's (1976) model in times of high stress, cognitive distortions are likely to become activated. As a result, dysfunctional thinking arises, which can make a person more vulnerable to the development of emotional as well as behavioural type psychopathology (e.g., Frey & Epkins, 2002). However, in the absence of stress, the dysfunctional cognitive structures, such as cognitive distortions, remain latent and do not directly lead to psychopathology. Nonetheless, the higher the number of underlying dysfunctional structures, the more vulnerable is a person to develop psychological symptoms, as several situations may trigger one of these dysfunctional thoughts (Beck, 2008).

According to several studies (e.g., Marques, Pereira, Barros, & Muris, 2013; Morris, Ciesla, & Garber, 2008), the association between cognitive distortions and psychopathology is valid for children as well as for adults. For example, anxiety sensitivity, anxiety control beliefs, and cognitive distortions have been found to be associated with each other (Weems, Costa, Watts, Taylor, & Cannon, 2007). Endorsement of cognitive distortions has also been identified in depressed adolescents (Kempton, Hasselt, Bukstein, & Null, 1994), in anxious youths (Watts & Weems, 2006), and in adolescents with both depressive and anxiety symptoms (Epkins, 2000). This relationship has been confirmed in both community and in clinical samples of children and adolescents (Schwartz & Maric, 2015). The importance of cognitive distortions has also been demonstrated in children with behavioural problems, such as hyperactivity symptoms (Flouri & Panourgia, 2011). Moreover, it is suggested that cognitive distortions can distinguish emotional from behavioural problems in community samples of adolescents (Epkins, 2000). These studies have also found that adolescents with emotional and comorbid behavioural and emotional problems reported more cognitive distortions than both groups with emotional problems only and control groups. No differences were found between the emotional problem group and the comorbid group, nor between the behavioural problem group and the control group. This surprising result appears to be of extreme importance because emotional and behavioural problems usually compromise a child's skills to effectively cope with developmental challenges and life stressors later on in life.

Grant et al.'s (2003) conceptual framework accounting for the role of stressors in child and adolescent psychopathology was used as a background to this study. In this model, stressors contribute to psychopathology, moderators influence the relationship between stressors and psychopathology, and mediators explain it. Moreover, these relations are characterised by a dynamic interaction. In other words, moderators (e.g., age and gender) are regarded as characteristics existing prior to the exposure to the stressor, with the particular feature of increasing or decreasing the probability that stressors will predict psychopathology. On the other hand, mediators (e.g., coping styles, as well as cognitive and family processes) are activated by stressors: they characterise the child or the child's environment in the presence of stressors. The child may sometimes possess some of the mediating characteristics before being exposed to the stressor, but the characteristic noticeably increases (or decreases) in response to the stressor. This study tested a mediation model.

Another important theoretical input was offered by Beck's theory (Beck, 1976) on cognitive distortions. Beck's model assumes that cognitive structures make a person more vulnerable to depression when the intensity of a stressor is heightened. Moreover, life adversity is more likely to lead to depression in individuals with maladaptive cognitions than in individuals without these cognitions. However, in the absence of life adversity, these dysfunctional cognitive structures remain latent and do not directly lead to mental health issues.

Finally, this investigation was designed to extend findings from another research study (Flouri & Panourgia, 2014) carried out during the academic year 2009–2010 and exploring, from a cross‐sectional point of view, the mechanisms through which maladaptive cognitions and difficulties in emotion regulation may explain the association between change in life adversity and emotional and behavioural problems in children. Children who took part in this previous investigation were followed‐up 1 year later (academic year 2010–2011) and assessed in a study in which a change mediation model was proposed. Longitudinal data were used to explore the mediating role of cognitive distortions in the relation between life adversity and emotional and behavioural problems. According to Muller, Judd, and Yzerbyt (2005), the mediation question focuses on the intervening mechanisms and processes that explain how an outcome is produced. In this study, life adversity contributed to psychopathology, whereas cognitive distortions explained this relationship.

### 1.1 This study

**Figure 1:**
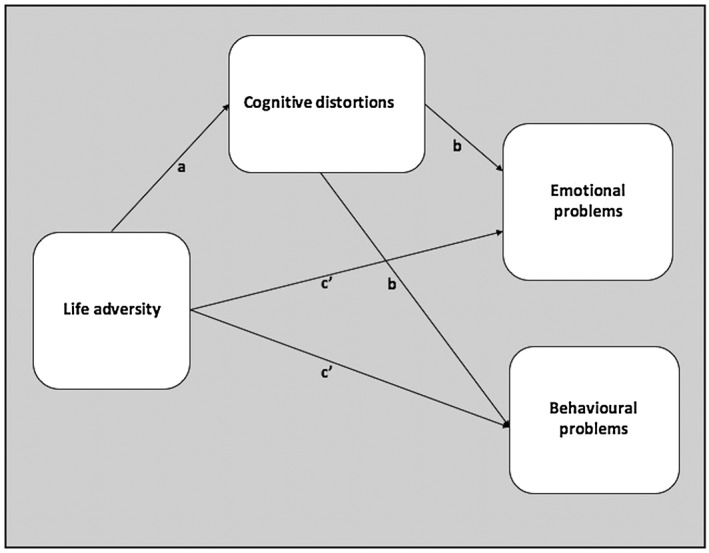
Path diagram for the mediation model tested in this study

Building upon, and also extending, findings from Flouri and Panourgia (2014) exploring whether cognitions could explain the relationship between life adversity and children's emotional and behavioural adjustment, the aim of this study was to assess these two separate, albeit related, dimensions of psychopathology. Emotional difficulties mainly refer to anxiety and depression, whereas behavioural problems, also known as antisocial issues, refer to acting out behaviours. These two dimensions were chosen on the basis of previous investigations (e.g., Hjemdal, Friborg, Stiles, Rosenvinge, & Martinussen, 2006; Phillips, Hammen, Brennan, Najman, & Bor, 2005) indicating the presence of behavioural and emotional symptoms in children and young people experiencing life adversity. This investigation was therefore aimed at testing whether an increase in cognitive distortions could account for the longitudinal relationship between the number of life adverse events and emotional and behavioural symptoms in a population of children (Figure 1 shows our mediation model). The longitudinal nature of this research allowed for the examination of the course of the same phenomenon over time, detecting factors associated with its development and thus providing the opportunity to draw causal inferences.

More specifically, this study tested the following hypotheses:

life adversity will be positively associated with emotional and behavioural problems;

the relationship between life adversity and emotional and behavioural problems will be mediated by cognitive distortions.

## 2 METHOD

### 2.1 Participants

This study followed up all participants (*N* = 430) of a cross‐sectional study conducted in three secondary schools in the South East of England during the academic year 2009–2010. All three schools were rated “satisfactory” by the Office for Standards in Education. The initial sample was above average in the proportion of adolescents who received free school meals and had special educational needs, but below average in the proportion of adolescents who spoke a first language other than English or were from a minority ethnic background. It consisted of 430 children (T1), but those with missing data on even one of the outcome variables (emotional, conduct, hyperactivity, and peer problems) were excluded from the final sample, resulting in 247 participants. Boys were 44.2%. Ages ranged from 12 to 16 years (*M* = 13.95, *SD* = 0.96). Of those with valid data, 47.8% answered affirmatively to whether they were living with both parents, 10.8% reported that they were on the school's Special Educational Needs register, 28.6% responded that they were eligible for free school meals, and 22.2% answered that they had been excluded from school for some reason or another. Most children (90.3%) were white British, and 91% reported English as their first language. Table 1 is a summary of the group's demographics.

**Table 1:** Descriptive statistics and pairwise correlations for all study variables

Variables	1	2	3	4	5	6	7	8	9	10	11	12	13	14
1. Life adversity (T2)														
2. Life adversity (T1)	0.24**													
3. Total difficulties (T2)	0.34**	0.22**												
4. Total difficulties (T1)	0.14*	0.41*	0.57**											
5. Cognitive distortions (T2)	0.27**	0.24**	0.54**	0.33**										
6. Cognitive distortions (T1)	0.12	0.26**	0.44**	0.57**	0.55**									
7. Age	0.08	−0.01	0.05	−0.05	−0.02	−0.15								
8. Gender (1 = boy and 2 = girl)	0.08	0.02	0.02	0.03	−0.02	0.03	0.03							
9. White British (1 vs. 0)	0.01	−0.06	0.08	−0.03	−0.04	0.08	0.04	0.19*						
10. English as first language (1 vs. 0)	0.05	0.02	0.03	0.01	−0.05	0.02	0.00	0.25**	0.85**					
11.Exclusion history (1 vs. 0)	0.06	0.05	0.20**	0.16*	0.10	0.11	0.17*	−0.24**	−0.05	−0.08				
12. Special educational needs (1 vs. 0)	0.11	0.08	0.05	0.12	0.06	0.12	−0.07	−0.13	−0.09	−0.16	0.10			
13.Free school meals (1 vs. 0)	0.11	0.09	0.26**	0.23**	0.10	0.22**	−0.01	0.03	−0.04	0.22**	0.15*	0.17*		
14. Two‐parent intact family (1 vs. 0)	−0.26**	−0.30**	−0.15*	−0.15*	−0.11	−0.18*	−0.14	0.06	0.04	0.11	−0.10	0.06	−0.24**	
*M*	4.62	11.03	13.27	14.21	57.32	59.84	13.95	1.56	0.90	0.91	0.22	0.11	0.29	0.48
*SD*	3.41	6.67	6.33	6.06	19.16	19.90	0.96	0.05	0.30	0.29	0.41	0.31	0.45	0.50
Ske.	1.16	0.86	0.42	0.30	0.40	0.25	−0.21	−0.23	−2.74	−2.90	1.35	2.55	0.95	0.09
Kur.	2.27	1.31	−0.28	−0.27	−0.03	−0.28	−0.40	−1.96	5.60	6.49	−0.18	4.55	−1.10	−2.01
Min‐Max	0–21	0–41	0–30	0–31	24–118	24–120	12–16	1–2	0–1	0–1	0–1	0–1	0–1	0–1

*Note*. *M* = sample mean or arithmetic average, Kur. = kurtosis, *SD* = standard deviation, Ske. = skewness.

* *p* < .05;

** *p* < .01 (two‐tailed).

### 2.2 Procedure

Participants were recruited in two ways. First, a letter of introduction and description of the study were sent to the three schools inviting their pupils to participate in a follow‐up study (T2). Telephone calls and personal contact from project staff were also made. Secondly, after the schools had agreed to take part, the parents of the children joining in the cross‐sectional study (T1) were contacted through a letter describing the procedure and the goals of the project. Parental consent was obtained for all adolescents joining in the follow‐up investigation, and ethical approval was given by the Faculty Research Ethics Committee of the Institute of Education (University of London). The time gap between T1 and T2 necessary to determine whether secondary school children were engaged in a biased way of thinking was 1 year. There are two key explanations for this relatively long‐term longitudinal investigation. First, because developmental changes in cognitive distortions were examined, something that does not develop rapidly over a brief time, a measurement interval of less than a year was unnecessary (Grammer, Coffman, Ornstein, & Morrison, 2013). Second, evidence from three further longitudinal studies (DuBois, Felner, Bartels, & Silverman, 1995; Hammen, 1988; Robinson, Garber, & Hilsman, 1995) suggests that negative perceptions of self in middle childhood and early adolescence can predict changes in depressive symptoms over a 6–12‐month period.

At the commencement of the questionnaire administration phase, the children were informed about the purpose of the study and assured that all collected data would be confidentially and securely held. They were also informed that their names would only be requested in order to allow the linkage of their data for the two time points and that at task completion, the page with their names would be destroyed. Their voluntary participation in the study was also emphasised, together with the opportunity to withdraw at any time and for any reason without needing to provide any kind of explanation. They were told that there would be no right or wrong responses and that leaving blank answers would be allowed. The children were given oral and written instructions describing the procedure. As preferred by the schools, the study questionnaires were completed under the supervision of teachers during regularly planned school lessons. All teachers received a letter describing the background of the study and a detailed protocol outlining the administration procedure.

### 2.3 Bias analysis

Of the original 430 children at T1, only 247 had complete data for all the outcome variables and were therefore included in this study's final sample. To determine whether subject attrition was random, we compared children included in the study's final sample to those discarded on all study variables, but no statistically significant differences were found. On average, children included in the final sample scored lower on emotional, hyperactivity, peer, and prosocial problems and slightly higher on conduct problems than those excluded. However, these differences were not statistically significant, *t*(248) = 0.45, *p* = .66; *t*(248) = 0.80, *p* = .42; *t*(249) = 0.44, *p* = .66; *t*(241) = 1.20, *p* = .23; *t*(247) = −0.08, *p* = .94, respectively. Moreover, children included in the final sample reported higher scores on the cognitive distortion measure and lower scores on the life adversity measure than the excluded ones, but these differences were not statistically significant, *t*(342) = −0.93, *p* = .35; *t*(241) = 0.50, *p* = .61, respectively.

Additionally, children in the final sample were more likely to be girls, to be eligible for free school meals, to have an exclusion history, to be on the Special Educational Needs register, and to live with both parents. Again, these differences were not statistically significant, χ^2^ (1) = 0.49, *p* = .48; χ^2^ (1) = 1.59, *p* = .21; χ^2^ (1) = 0.01, *p* = .91; χ^2^ (1) = 0.48, *p* = .49; χ^2^ (1) = 1.51, *p* = .22, respectively. There were no differences in ethnicity, χ^2^ (1) = 0.43, *p* = .51, or English as a first language, χ^2^ (1) = 0.10, *p* = .75, between the two samples.

### 2.4 Measures

*Background information or variables*: Information about school, age, gender, ethnicity, English as a first language, school exclusion history, family poverty (e.g., past or current eligibility for free school meals), special educational needs, and family structure was gained via means of a self‐report questionnaire. Family structure and ethnicity were coded into dichotomous variables (e.g., two‐parent intact or other and white British or other, respectively). This happened because not enough cases were present in these variables' categories, and detailed comparisons were therefore not possible. Previous life adversity (T1), previous emotional and behavioural problems (T1), and endorsement of cognitive distortions in the past (T1) were also included as background information or variables in this study. These variables were controlled for because they correlated with current life adversity (T2), current emotional and behavioural problems (T2), and current cognitive distortions (T2). Data on these variables were gained during the past academic year (2009–2010) and measured by the questionnaires described in the following sections.

*Current life adversity* was measured through the Adverse Life Events Scale (Tiet et al., 1998), which is a modified version of the Life Events Checklist (Coddington, 1972). The Life Events Checklist was developed by the National Centre for Post‐Traumatic Stress Disorder for diagnostic purposes and has acceptable validity (Johnson & McCutcheon, 1980) and test–retest reliability (Brand & Johnson, 1982). In particular, it has shown adequate test–retest reliabilities at 0.69 and 0.67 for a 3‐ and 7‐month interval for both negative and positive events (Coddington, 1984), respectively. In this sample, it showed low test–retest reliability (*r* = 0.46). However, we still decided to use exactly the same scale in order to measure changes in life adversity between Times 1 and 2 and to be able to compare the results of this study with previous findings.

The Adverse Life Events Scale is a 25‐item self‐report measure that was used at T1 and T2 to assess possible events occurring within a specified time frame. Each item describes events happening to parents, family or friends (e.g., “negative change in parent's financial situation,” “brother or sister left home,” and “close friend was seriously sick or injured”), or individual exposure to potential risky situations (e.g., “saw a crime or an accident”). Most of these events represent situations beyond young people's control (e.g., “someone in the family died,” and “someone in the family was arrested”). In this study, children were asked to report which events had happened to them within the past year. The 0–1 ratings for each item or life event were summed to provide a total score ranging from 0 to 25, with higher scores indicating higher levels of life adversity.

*Current emotional and behavioural problems* were assessed by the self‐report version of the Strengths and Difficulties Questionnaire (SDQ; Goodman, 1994, 1997), which was also used in T1. The SDQ comprises five dimensions: (a) hyperactivity (five items, e.g., “I am restless; I cannot stay still for long”), (b) emotional symptoms (five items, e.g., “I have many fears; I am easily scared”), (c) conduct problems (five items, e.g., “I am often accused of lying or cheating”), (d) peer problems (five items, e.g., “I get on better with adults than with people my own age”), and (e) prosocial behaviour (five items, e.g., “I try to be nice to other people. I care about their feelings”). Each item was rated following a 3‐point scale (ranging from 0 to 2): *certainly true*, *somewhat true*, and *not true*. A total difficulty scale score is calculated by summing the scores for hyperactivity, emotional symptoms, conduct problems, and peer problems. Scores for each scale range from 0 to 10 resulting in a total difficulties score from 0 to 40. Cutoff scores for the borderline or abnormal range (the SDQ cutoff score identifies 20% of the population) are 16 for total difficulties, six for emotional symptoms, four for conduct problems, six for hyperactivity, and four for peer problems, whereas the borderline or abnormal range for prosocial behaviour is 0–4 (www.sdqinfo.com).

The SDQ has shown good internal consistency (mean α = 0.73) and test–retest reliability over a 4‐ to 6‐month period (mean *r* = 0.62) and excellent cross‐informant correlation (mean *r* = 0.34; Goodman, 2001). In different countries, several investigations have documented satisfactory reliability and validity of the SDQ even though some studies have reported low reliability for conduct and peer problems (Muris, Meesters, & van den Berg, 2003). In this sample, internal consistency was high for the total difficulty scale (α = 0.83), acceptable for the peer problem scale (α = 0.66), and satisfactory for all other scales (α = 0.77 for the emotional symptoms, α = 0.70 for the conduct problems, α = 0.75 for the hyperactivity, and α = 0.70 for the prosocial behaviour).

*Current cognitive distortions* were assessed by Leitenberg, Yost, and Carroll‐Wilson's (1986) Children's Negative Cognitive Error Questionnaire (CNCEQ), which was also used in T1. The scale is comprised of 24 descriptions of hypothetical situations illustrating four types of cognitive distortions (catastrophising, overgeneralising, personalising, and selective abstraction) as emerging from Beck's model in three areas of a child's life (social, academic, and athletic). Each subscale of cognitive distortions comprises six items. Each item contains a vignette and a thought in response to that situation. Children were asked to read these hypothetical situations and rate, on a 5‐point scale, how likely they were to have had that thought, ranging from 1 (*not at all like I would think*) to 5 (*almost exactly like I would think*). The CNCEQ has proven acceptable internal consistency, test–retest reliability, and construct validity (e.g., Epkins, 2000). In this sample, internal consistency was high for total cognitive distortions (α = 0.96), catastrophising (α = 0.85), personalising (α = 0.86), and overgeneralising (α = 0.86). Cronbach's alpha was satisfactory for selective abstraction (α = 0.79).

## 3 RESULTS

### 3.1 Descriptive statistics

The study sample had experienced significant life adversity and was at risk for emotional and behavioural problems. More specifically, of those participants with valid data, only 19 (8.1%) described not having experienced any kind of life adversity over the past year. In total, the median for life adversity was four, ranging from 0 to 21. Moreover, children's scores on the SDQ were not in line with expected results in community samples (around 20% of a community sample is expected to score above SDQ cutoffs; Goodman, 1994, 1997). In particular, 23.1% of the study children were in the borderline or abnormal range for emotional symptoms, 33.2% for conduct problems, 38.9% for hyperactivity, 18.3% for peer problems, 26.4% for prosocial behaviour problems, and 34.4% for total difficulties. Finally, children in the final sample tended to report, on average, high levels of cognitive distortions, the mean was 57.32 (*SD* = 19.16), and the median was 57. Table 1 presents descriptive statistics for all study variables and their zero‐order correlations.

### 3.2 Analytic strategy

Overall, 3.2% of values were missing, and between 10.9 and 36.8% were missing at variable level. Little's (1988) chi‐square statistic showed that missing data were completely random (χ^2^_MCAR_ = 3361.16, *df* = 2011, *p* < .001). Therefore, we imputed missing data on the covariates using multiple imputation in SPSS 18 (Graham, Olchowski, & Gilreath, 2007, for a review of the benefits of using multiple imputation methods). We produced five imputed datasets using the Markov Chain Monte Carlo procedure to account for the uncertainty in imputed data.

This study's mediation hypothesis was tested by fitting structural equation models in Mplus 6.1 (Muthén & Muthén, 2010). By following this approach, we were able to test how well a process model, explaining the relationship between a predictor variable X to an outcome variable Y via an intervening process, fits the observed data (Hayes, 2009). In particular, Mplus provided model fit and parameter estimates based on the five imputed datasets, correcting for the uncertainty introduced by the imputations. Four indices of model fit were employed to indicate good fit: (a) the root mean square error of approximation (RMSEA) and (b) the standardized root mean square residual (SRMR) values, both of which were less than .05, (c) the Tucker‐Lewis index (TLI) and (d) comparative fit index (CFI) values, which were close to 1.00 (Chen, Curran, Bollen, Kirby, & Paxton, 2008).

For us to estimate simple mediation, the bootstrap method recommended by Preacher, Zhang, and Zyphur (2011) was used and ran in each of the five imputed datasets in Mplus. Bootstrap confidence interval (CI) is considered as one of the best approaches to examine the indirect effect (MacKinnon, Lockwood, Hoffman, West, & Sheets, 2002) and was preferred over the Sobel test or causal steps approach because of the small sample size. Therefore, by following this nonparametric resampling procedure for testing indirect effects, higher power and better control over Type I error were obtained (MacKinnon et al., 2002). In this specific analysis, the estimates were based on 5,000 bootstrap samples. In Mplus significance tests for the indirect effect of X on Y via the mediator variable and bias‐corrected bootstrap, CIs can be obtained. However, in the case of multiple imputed data sets, CIs are not usually generated (Preacher et al., 2011).

### 3.3 Confirmatory factor analysis

First, before fitting the structural equation models, we conducted a confirmatory factor analysis to derive the latent constructs of emotional and behavioural problems and cognitive distortions (both at T1 and T2).

#### 3.3.1 Emotional and behavioural problems

Latent constructs of emotional, peer, conduct, and hyperactivity difficulties (both at T1 and T2) were created by the items of the perceived emotional, peer, conduct, and hyperactivity difficulty scales. Secondly, this first‐order latent factor solution was compared to two second‐order latent factor solutions. The first of these factors was emotional problems created by emotional and peer difficulties and behavioural problems created by conduct and hyperactivity difficulties. The second of these factors was total difficulties created by emotional, peer, conduct, and hyperactivity difficulties. Then, the first‐order latent factor solution was compared to a third‐order latent factor solution (total difficulties by emotional and behavioural problems). The model that best fitted the data was the one in which emotional problems loaded on emotional and peer difficulties and behavioural problems loaded on conduct and hyperactivity difficulties. However, the fit was rather poor (RMSEA = 0.05; CFI = 0.89; TLI = 0.87; and SRMR = 0.06 for emotional and behavioural problems at T1, and RMSEA = 0.08; CFI = 0.81; TLI = 0.79; and SRMR = 0.08 for emotional and behavioural problems at T2). Model fit improved considerably when these analyses were replicated by parcelling items (Bandalos, 2002). 1Item parcelling is a procedure to combine individual items typically used in CFA or SEM. Parcels are an alternative to using the individual items and are usually created by taking the sum or mean of a set of items within a factor. Again, the model with the best fit was emotional problems loaded on emotional and peer difficulties and behavioural problems loaded on hyperactivity and conduct difficulties (RMSEA = 0.05; CFI = 0.94; TLI = 0.92; and SRMR = 0.05 for emotional and behavioural problems at T1, and RMSEA = 0.06; CFI = 0.94; TLI = 0.92; and SRMR = 0.05 for emotional and behavioural problems at T2).

#### 3.3.2 Cognitive distortions

Latent constructs of cognitive distortions (both at T1 and T2) were subsequently created using the items constituting those very same cognitive distortions. This first‐order latent factor solution—for cognitive distortions at both T1 and T2—was compared to another first‐order latent factor explanation (overgeneralising, by the items of the perceived overgeneralising scale; catastrophising, by the items of the perceived catastrophising scale; personalising, by the items of the perceived personalising scale; selective abstraction, by the items of the perceived selective abstraction scale). Moreover, it was equated to a second‐order latent factor solution (total cognitive distortions by overgeneralising, catastrophising, personalising, and selective abstraction). Once again, model fit considerably improved when these analyses were replicated by parcelling items (Bandalos, 2002). The model that best fitted the data was cognitive distortions (at both T1 and T2) loaded on the items of the perceived cognitive distortions (RMSEA = 0.09; CFI = 0.98; TLI = 0.96; and SRMR = 0.03 for T1 cognitive distortions, and RMSEA = 0.12; CFI = 0.96; TLI = 0.93; and SRMR = 0.03 for T2 cognitive distortions). RMSEA fit deteriorated when item parcelling was applied, whereas all the other measures of model fit improved. RMSEA is considered to be relatively independent of sample size (Hooper, Coughlan, & Mullen, 2008). On the other hand, there is evidence supporting the fact that RMSEA over rejects true models when sample size is less than 250, which was the case here. In this study, SRMR is suggested as the preferred measure (Chen et al., 2008).

### 3.4 Structural equation models

In the first step of the mediation model, T2 emotional problems and T2 behavioural problems (outcome variables) were regressed on T2 life adversity (predictor variable) adjusting for T1 life adversity, T1 emotional problems, T1 behavioural problems, the two dummies for school (with School 2 as reference), and all control variables (age, gender, ethnicity, special educational needs, exclusion history, free school meals, English as first language, and two‐intact parent family). T2 life adversity was found to be positively associated with T2 behavioural problems (*b* = 0.03, *SE* = 0.01, *p* = .009) only. T2 life adversity did not predict T2 emotional problems and thus mediation analysis could not be carried out. Moreover, as can be seen in Table 2, T1 behavioural problems significantly accounted (*b* = 0.80, *SE* = 0.15, *p* < .001) for T2 behavioural problems. Children at School 1 and children with an exclusion history reported higher levels of behavioural problems at T2 (*b* = 0.19, *SE* = 0.06, *p* = .004; *b* = 0.15, *SE* = 0.08, *p* = .049, respectively), whereas special educational needs were negatively associated with T2 behavioural problems (*b* = −0.22, *SE* = 0.10, *p* = .030). The fit of this model was good (CFI = 0.93; TLI = 0.92; RMSEA = 0.03; SRMR = 0.07).

**Table 2:** Results of Structural equation modelings

Steps	Outcome variables	Predictor variables	*b*	*SE*
Step 1	Emotional problems	Life adversity (T2)	0.02	0.01
	(Time 2)	Life adversity (T1)	−0.01	0.01
		Emotional problems (T1)	0.74**	0.25
		School 1	−0.04	0.07
		School 3	0.08	0.06
		Age	0.03	0.03
		Girl	0.10	0.06
		English as first language	−0.08	0.15
		Exclusion history	−0.14*	0.07
		Special educational needs	0.07	0.09
		Free school meals	0.07	0.07
		Two‐parent intact family	−0.01	0.06
		White British	0.11	0.14
	Behavioural problems	Life adversity (T2)	0.03**	0.01
	(Time 2)	Life adversity (T1)	−0.00	0.01
		Behavioural problems (T1)	0.80***	0.15
		School 1	0.19**	0.06
		School 3	0.09	0.06
		Age	0.01	0.03
		Girl	−0.08	0.07
		English as first language	0.13	0.11
		Exclusion history	0.15*	0.08
		Special educational needs	−0.22*	0.10
		Free school meals	0.04	0.08
		Two‐parent intact family	0.08	0.06
		White British	0.03	0.12
Step 2	Cognitive distortions	Life adversity (T2)	0.04***	0.01
	(Time 2)	Life adversity (T1)	0.00	0.01
		Cognitive distortions (T1)	0.57***	0.09
		School 1	0.11	0.11
		School 3	0.16	0.10
		Age	0.04	0.05
		Girl	−0.05	0.09
		English as first language	0.00	0.24
		Exclusion history	0.03	0.13
		Special educational needs	−0.11	0.15
		Free school meals	−0.05	0.11
		Two‐parent intact family	0.07	0.09
		White British	−0.08	0.23
Step 3	Behavioural problems	Life adversity (T2)	0.02	0.01
	(Time 2)	Life adversity (T1)	−0.00	0.01
		Cognitive distortions (T2)	0.18***	0.06
		Cognitive distortions (T1)	−0.09	0.06
		Behavioural problems (T1)	0.85***	0.17
		School 1	0.18**	0.06
		School 3	0.07	0.06
		Age	0.01	0.03
		Girl	−0.05	0.06
		English as first language	0.11	0.13
		Exclusion history	0.15*	0.07
		Special educational needs	−0.20*	0.10
		Free school meals	0.03	0.09
		Two‐parent intact family	0.07	0.05
		White British	0.06	0.12

* *p* < .05.

** *p* < .01.

*** *p* < .001.

Following this, in Step 2 (Table 2), T2 cognitive distortions (mediator variable) were regressed on T2 life adversity (predictor variable) controlling for all control variables (T1 cognitive distortions, T1 life adversity, the two dummies for school [with School 2 as reference], age, gender, ethnicity, special educational needs, exclusion history, free school meals, English as first language, and two‐intact parent family). In Step 3 (Table 2), T2 behavioural problems (outcome variable) were regressed on both T2 life adversity (predictor variable) and T2 cognitive distortions (mediator variable). The model also adjusted for all control variables. In these steps (2 and 3), path *a*, path *b*, and the indirect effect were estimated and a significance test for the indirect effect of T2 life adversity on T2 behavioural problems via T2 cognitive distortions was requested and provided.

Table 2 displays the aforementioned results. T2 life adversity and T1 cognitive distortions predicted T2 cognitive distortions. T2 life adversity was not a significant predictor of T2 behavioural problems. On the other hand, T2 cognitive distortions predicted a significant amount of variance in Time 2 behavioural problems. In addition, having behavioural problems in the past (Time 1), attending school 1, having an exclusion history, and not being listed on the Special Needs register significantly predicted behavioural problems in T2. Finally, the indirect effect of Time 2 life adversity on T2 behavioural problems via T2 cognitive distortions was statistically significant (*b* = 0.01, *SE* = 0.00, *p* = .007), suggesting that change in cognitive distortions mediated the relationship between change in life adversity and change in behavioural problems. This model fitted the data well (RMSEA = 0.02; CFI = 0.98; TLI = 0.98; SRMR = 0.06).

## 4 DISCUSSION

This study was carried out with the participants recruited in another investigation (Flouri & Panourgia, 2011), who were followed‐up after a year from baseline assessment. This investigation was designed to explore whether cognitive distortions could explain the longitudinal relationship between life adversity and children's emotional and behavioural adjustments. In other words, it explored whether an increase in cognitive distortions could explain why an increase in life adversity was related to an intensification in emotional and behavioural problems. Because of its longitudinal nature, this study examined the course of the same occurrence over time, detecting factors associated with its development and thus providing the chance to reach causal interpretations (Lutz & Hill, 2009).

Even though research has shown a solid relationship between cognitive distortions and emotional problems (Schwartz & Maric, 2015), one of the most important results of this investigation was that change in life adversity had no impact on change in emotional problems. There may have been a different outcome (a) had we considered specific aspects of emotional problems (e.g., emotional symptoms and peer difficulties) in the study's change models and (b) had we used information obtained by parents, which has been shown to have more predictive value than self‐report data (Goodman, Meltzer, & Bailey, 2003). Contrary to this, a surge in life adversity was found to be associated with an increase in cognitive distortions. This was, in turn, linked to an intensification in the number of behavioural problems. Therefore, change in cognitive distortions acted as a mediator between change in life adversity and change in behavioural problems. When life adversity alterations could be observed, changes in children's behavioural problems were also likely to occur. This was mainly due to changing as well as negatively biased interpretations given by participants to specific life events.

Secondly, this study confirmed the results of previous investigations on the key role played by cognitive distortions in the areas of adjustment and well‐being (Morris et al., 2008). As for adults, and in accordance with previous findings (Epkins, 2000), in high‐risk children, cognitive distortions were found to be positively associated with mental health problems and particularly conduct problems. Moreover, although the specific content of the aforementioned cognitive distortions was not examined, a change mediation model was proposed. Future studies linking life adversity to children's emotional and behavioural problems in a longitudinal way should examine outcome specificity as well as specificity in the cognitive mechanisms underlying psychological adjustment against adversity.

Cognitive distortions at T2 (2010–2011) were measured by the CNCEQ (Leitenberg et al., 1986) because this was used at T1 (2009–2010). Although this measure is able to assess a variety of cognitive constructs linked to depression (Leitenberg et al., 1986) and anxiety problems in young people (Watts & Weems, 2006; Weems et al., 2007), it lacks specificity. Future researchers should consider the use of the CNCEQ‐R (Maric, Heyne, van Widenfelt, & Westenberg, 2011), a tool developed to address the overlap between different cognitive distortion categories and specificity of cognitive distortions (e.g., “mind reading” and “underestimation of the ability to cope”), which are differentially related to anxiety symptoms (e.g., Epkins, 2000; Weems et al., 2007).

Moreover, because emotional and behavioural issues are characterised by a certain cognitive aspect involving specific cognitive deficiencies and distortions, we recommend that future investigations explore cognitive distortions as well as other cognitive deficits (Dodge & Schwartz, 1997). For instance, hyperactivity has been found to rely on cognitive deficiencies and not on distortions (Kendall & MacDonald, 1993). Similarly, conduct problems have been found to be related to thought shortages and distortions alike (Lochman & Dodge, 1998). Additionally, the emergency and use of cognitive resources may be affected by issues related to cognitive development. Because this pathway may not be replicated in younger children, the results of this study would benefit from being compared to other findings deriving from investigations involving younger at‐risk children, with the general goal of designing age‐specific cognitive interventions.

The above conclusions should be evaluated in the light of several limitations. First of all, most children came from socioeconomically disadvantaged backgrounds and were at high‐risk in terms of life adversity and mental health issues. Secondly, it would be advisable for future studies to examine whether findings can be replicated in longitudinal analyses using longer time periods. Thirdly, life adversity could only be tested as the outcome of mental health problems rather than as a risk factor per se. Fourthly, information from parents, caretakers, and teachers was not taken into account due to practical issues of access such as time commitment. Therefore, the main variables of this study were reported by the same informants, namely, the children, allowing for shared variance problems. Thus, reliance on single‐informant measurement strategy could have influenced the accuracy of responses.

Future studies could attempt to use multiple informants considering evidence that teachers may be reliable informants of conduct and hyperactivity problems and parents of emotional problems (Achenbach & Rescorla, 2001). Finally, the level of missing data was quite high due to (a) low levels of nonverbal and verbal cognitive abilities, which were required to complete the questionnaires; (b) questionnaires administration time set during the afternoon, a time of the day in which participants could have felt bored and tired; (c) absence of researchers at the time of questionnaires administration; and (d) the rate at which some of the students had already left the schools in question. All teachers and teaching assistants were asked to follow a detailed protocol outlining the administration procedure. Nevertheless, this could not ensure that teachers followed procedures in the very same way

All the above mentioned findings are significant because they offer valuable recommendations to policy makers, as well as to clinicians and researchers alike. In this study, an increase in life adversity was found to be a strong predictor of an escalation in behavioural problems. By reducing the number of life adversities over time, at‐risk children could be prevented from further developing behavioural problems. Furthermore, study results identified a specific source for prevention strategies. The tested model suggested that an increase in cognitive distortions was likely to represent the link between an increase in life adversity and an intensification in behavioural problems in children. These outcomes could aid clinicians understand children's preeminent behavioural responses to increased levels of adversity.

This investigation also provided information regarding the influence of time on the relationship between life adversity and behavioural issues, as well as on the mediating effect of cognitive distortions. Treatments should ideally be focused on changing the number of adverse life experiences in order to increase behavioural adjustment over time. Results also highlighted the importance of challenging and minimising children's cognitive distortions, with the more general goal of developing interventions that may help at‐risk children and young people function adaptively despite mounting adversity.

## CONFLICT OF INTEREST

The authors have declared that they have no conflict of interest.
